# A scientometric review of genome-wide association studies

**DOI:** 10.1038/s42003-018-0261-x

**Published:** 2019-01-07

**Authors:** Melinda C. Mills, Charles Rahal

**Affiliations:** University of Oxford and Nuffield College, New Road, Oxford, OX1 1NF UK

## Abstract

This scientometric review of genome-wide association studies (GWAS) from 2005 to 2018 (3639 studies; 3508 traits) reveals extraordinary increases in sample sizes, rates of discovery and traits studied. A longitudinal examination shows fluctuating ancestral diversity, still predominantly European Ancestry (88% in 2017) with 72% of discoveries from participants recruited from three countries (US, UK, Iceland). US agencies, primarily NIH, fund 85% and women are less often senior authors. We generate a unique GWAS H-Index and reveal a tight social network of prominent authors and frequently used data sets. We conclude with 10 evidence-based policy recommendations for scientists, research bodies, funders, and editors.

## Abstract (web summary)

Melinda Mills and Charles Rahal discuss genome-wide association studies published in the last 13 years, finding increases in sample sizes, rates of discovery, and traits studied over time. They discuss limitations, including sample diversity, and make recommendations for scientists and funding bodies.

## Introduction

Since the human genome was first sequenced in 2003, almost 3700 genome-wide association studies (GWAS) have agnostically identified thousands of genetic risk variants and their biological function^1–3^. Unlike Mendelian disorders caused by a single genetic defect, most complex diseases such as diabetes or coronary heart disease rely on multiple genetic variants and their exposure to—and interaction with—social and environmental factors. Contemporary GWAS combine data from participants across multiple data sets in the form of a meta-analysis^4^ to analyze millions of these variants. Discoveries have led to clinical findings from diseases such as breast cancer and Alzheimer’s to anthropometric and behavioral traits, with momentum moving from the study of association to biological function^5^.

Although excellent narrative reviews document the scientific contributions of GWAS^1,2,5,6^, there has been no systematic scientometric study as yet. Such a study is crucial for researchers, data providers, editors, and consortiums working in this area to understand the strengths and potential gaps in current research and is essential to plan future investments in data collection and science policy for funders, research bodies, and national governments. Furthermore, research evaluations and funding exercises increasingly rely on scientometric rankings of author productivity, such as the *H*-index to measure the productivity and impact of scientists. Funders and national governments also strive to find useable metrics to trace the impact and usage of their scientific investments (such as large data collection infrastructures or investment in scientists). They also endeavor to measure whether policies to enact change have been realized. This includes initiatives such as the National Institute of Health’s (NIH) Revitalization Act, for instance, which mandates the inclusion of minorities as subjects. Most funding bodies and Universities have likewise noted lower levels of women and ethnic minorities in senior biomedical positions and implemented policies to counteract these trends, but there are limited metrics for evaluation across the genomic landscape^7–9^.

We first study participant demographics, sample sizes, ancestry, the geographic distribution of participant recruitment, the number and *p* values of genetic associations, journal diversity, and disease focus. We draw on over 13 years of GWAS discoveries (March 2005 to October 2018) from the NHGRI-EBI GWAS Catalog (hereafter, the Catalog) produced by the US National Human Genome Research Institute (NHGRI) in conjunction with the European Bioinformatics Institute (EBI)^10,11^. We then link the Catalog to external PubMed and United Nations (UN) population data and manually curate the most frequently used data sets, which cover over 85% of all GWAS by cumulative sample size across approximately a third of all papers. We rank and map top funders by ancestry and disease, isolate key consortiums, engage in an analysis of gender and authorship, create a unique GWAS *H*-Index and undertake a social network analysis of author centrality. This unique overview allows us to formulate 10 concrete evidence-based policy recommendations. Our accompanying, Supplementary Methods and Supplementary Note 1 describe the methods and data used to produce the results and dynamically pull in new data, which will regularly update our analyses, creating an open, live database over time.

## Sample sizes, associations found, diseases studied, and journal diversity

**Fig. 1:**
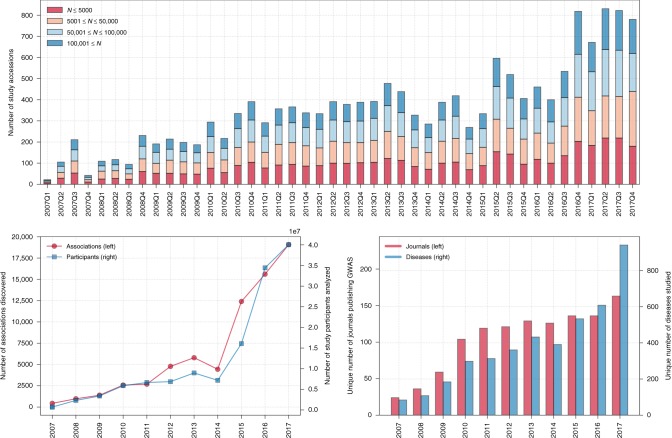
The growth of GWAS, 2007–2017. The upper panel shows the number of study accessions published per quarter over time colored according to sample size to show the growth of larger (100,001 ≤ *N*) GWAS. The lower left panel shows the strong positive correlation between the number of associations found and the number of participants used in GWAS over time. The lower right panel shows the growth in the number of unique traits examined as well as the number of unique journals publishing GWAS over time. 2007–2017 is selected since only 10 entries occurred before 2007. Each panel contains full calendar years only. Source: NHGRI-EBI GWAS Catalog

Figure 1 shows the explosion in GWAS research since 2007. Although the first entry within the GWAS Catalog is dated 10 March 2005, only 10 entries were made in 2005 and 2006. A major breakthrough occurred in 2007, with a widely heralded paper published by the Wellcome Trust Case Control Consortium^12^, later termed a masterwork of diplomacy owing to the aggregation of the data involved^6^. As of 29 October 2018, the Catalog records 3639 individual research papers, which span 5849 unique Study Accessions (unique identifiers ascribed to studies of specific traits within a paper) across 3508 unique diseases/traits, which map to 2532 unique Mapped Experimental Factor Ontology traits. The average number of associations or hits per study is 15.3, with an average *p* value of 1.3729 × 10^–6^. Only 49,451 out of 89,588 (55.20%) reported associations meet the heralded *p* ≤ 5×10^–8^ threshold, with most remaining within or below the borderline level, with recent work suggesting a possible relaxation in the current threshold^13^. *Nature Genetics* has been the most frequent publisher over time, although in 2017, GWAS were most frequently published by *Nature Communications*. At the time of writing, the largest study in the Catalog presently contains 1,030,836 subjects.

## Ancestral diversity, geographical concentration, and data sets used

Considerable attention has been paid to the disparities underlying the ancestral diversity of study participants for technical reasons such as population stratification^14^, reduced linkage disequilibria^15^, genetic diversity and admixture^16^, cultural distrust and social misuses, and interpretations^17,18^. Including diverse participants is crucial for understanding genetic heterogeneity in disease phenotypes and the creation of an equitable distribution of personalized medicine^19^. There is also a limited portability of polygenic scores across populations, which we return to in our final discussion^20^.

**Fig. 2:**
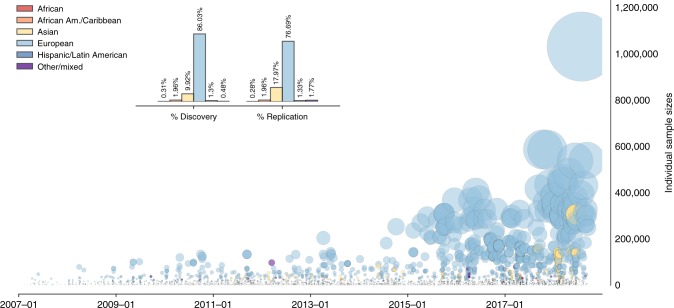
GWAS Participant Ancestry over Time, 2007–2017. The main panel shows a disaggregation of our broad ancestral categories field, which is a direct mapping from the 17 broad ancestral categories identified in the Catalog. We drop all rows where any proportion of the ancestry is not recorded, and for combinations of ancestries (e.g., European and African) we create a new field: Other/Mixed. The inset aggregates this across the entire sample but partitions the data across discovery and replication phases. 2007–2017 is selected since only 10 entries occurred before 2007 and we have complete information for the year 2017. Source: NHGRI-EBI GWAS Catalog and author mapping

Figure 2 visualizes a customized Broader Ancestral Category^21^ field, which subsumes hundreds of combinations of seventeen different broad ancestral categories mapped to seven unique broader categories. Our results (when dropping rows of the Catalog that contain any unrecorded ancestries) concur with existing estimates^21,22^, showing that on aggregate, ancestry in genetic discovery has been highly unequal and dominated by participants of European ancestry (86.03% discovery, 76.69% replication, 83.19% combined). Other prominently studied ancestries are Asian (9.92% discovery, 17.97% replication, 12.37% combined), African American or Afro-Caribbean (1.96% discovery, 1.96% replication, 1.96% combined), Hispanic or Latin American (1.30% discovery, 1.33% replication, 1.30% combined), Other or Mixed (0.48% discovery, 1.77% replication, 0.87% combined) and African (0.31% discovery, 0.28% replication, 0.30% combined) ancestry. Table 1 shows that the percent per annum of European ancestry samples fluctuates considerably and has been as high as 90.76% in 2016 and as low as 71.98% in 2012. In 2008, not a single study utilized participants of African ancestry. By partitioning the data into discovery and replication samples, we show that the percent of European ancestry samples used for initial discovery is substantially higher than for replication, and that samples of Asian ancestry make up a considerably higher share of replications than for initial discovery.

**Table 1:** Percent of broader ancestry Group in GWAS over time, 2007–2017

Year	Ancestry					
	European	Asian	African	Hispanic/Latin American	Other/Mixed	African American or Afro-Caribbean
2007	95.47	2.14	0.01	0.72	1.18	0.49
2008	95.29	2.95	0	0	1.22	0.54
2009	88.17	7.10	0.26	0.22	3.36	0.88
2010	86.85	9.89	0.27	0.06	2.44	0.49
2011	78.23	15.82	0.16	0.4	1.71	3.68
2012	71.98	19.47	0.31	0.88	2.87	4.48
2013	82.20	11.69	0.39	0.79	0.62	4.32
2014	76.61	18.62	0.25	1.15	0.98	2.4
2015	87.81	9.43	0.28	0.77	0.53	1.18
2016	90.79	4.65	0.17	1.47	1.10	1.83
2017	87.96	6.33	0.57	1.2	0.67	2.13

A disaggregated temporal breakdown of our synthetic Broader ancestry field. Any ancestries that are in any part not recorded are dropped. All numbers are percentages. 2007–2017 is selected since only 10 entries occurred before 2007 and we have complete information for the year 2017

A regular expression-based exercise to extract information from the free text related to discovery and replication sample descriptions identifies 212 and 150 unique terms, respectively for classifying participants in terms of their race, region, country, ethnicity, or ancestry. This ranges from the most common term of European, to hybrid terms such as Caucasian Eastern Mediterranean along with multiple other examples of polyvocality. Our accompanying replication material provides a more empirically transparent and rigorous evidence base compared with previous research that reported that around a fifth of papers use classification schemes in logically ambiguous ways^23^ and estimates that there were up to 26 terms to describe participants of African ancestry^22^.

This decomposition of the free text field also allows us to examine categorizations of Native or Indigenous populations. These groups have had a particularly complex relationship with genomics research, but have also revealed some key genetic associations^17,24^. Our analysis shows eight terms that explicitly use nomenclature related to Native, Indigenous, or Aboriginal populations, such as Aboriginal Canadian (a term seen twice, 15 observations in total), Martu Australian Aboriginal (a term seen thrice, 752 observations in total) or various terms related to Native Hawaiians (a term seen 11 times, 3179 observations in total) and that they contribute 0.006% of all samples used (with the term Native Hawaiian used most frequently, and Alaska Natives mentioned thrice). When using a curated lookup table based on the United Nations Declaration on the Rights of Indigenous Peoples (to include terms such as Pima Indians)^25^, this number increases to 0.022%.

**Fig. 3:**
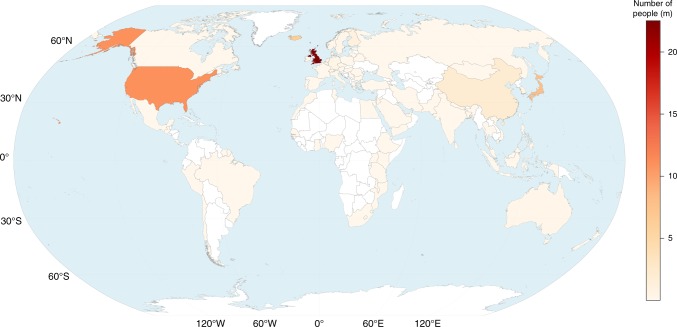
A Choropleth Map of the Concentration of GWAS Participant Recruitment. A choropleth map (Robinson projection) detailing the geographic recruitment of GWAS participants. Source: NHGRI-EBI GWAS Catalog, Natural Earth (v4.0.0) and the CIA World Factbook. Replication material provides a per-capita population adjusted version

Uniquely, we also provide the first systematic breakdown of recruitment of GWAS subjects by examining the Country of Recruitment field^21^ provided by the Catalog for studies where only a single country was recruited from (Fig. 3). We show that 71.80% of participants are recruited from only three countries; the US, UK, and Iceland. Although participants from the United States are most frequently the basis for the largest number of studies (41.01% of all studies), the United Kingdom dominates in terms of the number of participants (40.50% of all participants) analyzed. Conversely, although 1.13% of recorded studies involve Icelandic participants, the small Icelandic population (around 334,000) represents 11.52% of all participants contributed to GWAS research. In terms of the ratio of the number of observations contributed by a country relative to the population of the country^26^, Iceland is by far the largest (19.13), followed by the United Kingdom (0.32). Note that owing to the way in which data on recruitment from multiple countries is curated, these numbers can only be used to compare between countries, rather than in absolute terms. This result is predominantly driven by data from deCODE genetics, a major biotech company founded in 1996 in Reykjavík, Iceland. Aggregating to the continental level, Table 2 illustrates a similar but distinct global picture of genomic research: European countries contribute 58.54% of recruited participants and North America a further 19.99% (29.09% and 42.57% of all studies, respectively).

**Table 2:** Breakdown of GWAS participants by top countries and continents

Country	Continent	Count	*N*	Count (%)	*N* (%)	Per Rec
United Kingdom	Europe	662	22521698	10.54	40.50	0.34
United States	North America	2576	10997635	41.01	19.78	0.03
Japan	Asia	481	7940622	7.66	14.28	0.06
Iceland	Europe	71	6409109	1.13	11.52	19.13
China	Asia	500	2059693	7.96	3.70	0.00
Finland	Europe	218	1193333	3.47	2.15	0.22
South Korea	Asia	256	857072	4.08	1.54	0.02
Netherlands	Europe	175	663477	2.79	1.19	0.04
Germany	Europe	175	434824	2.79	0.78	0.01
Australia	Oceania	110	320458	1.75	0.58	0.01

Catalog’s Country of Recruitment field cleaned and aggregated to continent level (CIA World Factbook definitions). The Per Rec field relates to number of observed recruitments (with overlap) divided by 2017 UN population estimates. The time period is all studies from 2007 to 2017

We manually extracted a list of the most frequently used datasets (sometimes referred to cohorts) across the majority of the largest 1250 GWAS as of 29 August 2018, with the objective of providing the first systematic estimate of the frequency and identification of data sources used in GWAS (Table 3). The most frequently used data sets have several key distinguishing features^27^. First, echoing our geographic analysis, frequently used data are from industrialized countries (Netherlands, US, UK, Ireland, Germany, Iceland), which share similar rates of disease prevalence and population profiles. Second, most engaged in random probability or population sampling to gain as representative a sample as possible, something that is not characteristic of emerging large data sets such as the healthy, older and higher socioeconomic status participants in the UK Biobank^28^ or direct-to-consumer genetic data. Third, they are cohorts that are deeply and richly phenotyped across many diseases, future-proofing them for multiple needs. Fourth, many are older populations with disease diagnosis aimed at unraveling the pathways to disease and disability in old age. In this respect, they miss the longer-term development of disease and intervention possibilities that an asymptomatic younger population might afford (except for the 1958 British Birth Cohort or additional data collection in cohorts such as the FHS). Fifth, they are all prospective longitudinal data sets, following individuals or birth cohorts over a longer period, thus facilitating a life-course approach to understanding the pathways to certain diseases, disability, and mortality. Sixth, all but one of these cohorts is comprised of predominantly female participants (ranging from 48 to 100%). This sex ratio imbalance is rarely addressed, yet sexual dimorphism or sex differences in disease are highly relevant^29,30^. Finally, although many started as focused hypothesis-driven clinical samples to study one type of disease, most have expanded to contain a breadth of phenotypes and document a trend of adding new samples or generations over time.

**Table 3:** Most frequently utilized data sets across the largest GWAS

Cohorts	Count	*N*	Country of recruitment	Ager range	Study design	Female (%)
Rotterdam Study (RS)	398	14,926	Netherlands	55–106	Prospective cohort	57
Cooperative Health Research in the Region of Augsburg (KORA)	255	18,079	Germany	24–75	Population-based	50
Framingham Heart Study (FHS)	207	15,447	US	5–85*	Prospective cohort, three generation	54
Atherosclerosis Risk in Communities Study (ARIC)	204	15,792	US	45–64	Prospective cohort, Community	55
Cardiovascular Health Study (CHS)	179	5888	US	65+	Prospective cohort	58
British 1958 Birth Cohort Study (1958BC/NCDS)	156	17,634	UK	0+	Prospective birth cohort	48
UK Adult Twin Register (TwinsUK)	140	12,000	UK, Ireland	18–97	Longitudinal entry at various times	84
European Prospective Investigation into Cancer CANCER (EPIC)	132	521,330***	10 EU countries	21–83**	Prospective cohort	71
Nurses Health Study (NHS)	129	121,700	US	30–55	Prospective cohort	100
Study of Health in Pomerania (SHIP)	127	4308	Germany	20–79	Prospective cohort	51

The top 10 most frequently utilized cohorts across the majority of the largest third of all GWAS studies as of 29 August 2018 (with studies ranked by the number of times they are involved in a GWAS), manually extracted and harmonized. Additional fields (country of recruitment, age range, and study design) manually curated from web searches. * denotes originally 30–62 years, ** denotes variation by country, *** denotes full sample, including non-genotyped participants

## GWAS researchers: impact, networks, and gender bias

In total, we estimate that there have been 122,141 authorship contributions made by 39,893 unique authors. GWAS meta-analysis has traditionally involved a collaboration of many authors contributing a data set or expertize, with 33.71 authors on average per paper returned from the PubMed database. The highest number of authors on one paper is 559, who collaborated on a study of type 2 diabetes and metabolic traits^31^.

Table 4 shows the 10 authors with the highest score in our newly derived GWAS *H*-Index (Supplementary Methods), which goes beyond a standard H-Index to estimate the importance, significance and impact of a scientist’s cumulative GWAS-related research contributions (the replication material outlined in Supplementary Note 1 provides a full ranking of all authors who have been involved in more than one GWAS and have more than 10 citations). These key authors share several striking traits. Many (Stefánsson, Thorsteinsdóttir, and Thorleisfsson) are from deCODE Genetics; pioneers in terms of large sample size, detailed genetic and medical information and the development of new statistical tools. The upper realms of the table also feature key academics at the center of prominent data sets such as Uitterlinden, Hofman, van Duijin, and Rivadeneira, who are key investigators of The Rotterdam Study and Generation R Study. In a recent *Nature* article describing hyperprolific authors, Uitterlinden provides a candid explanation of his authorship. In addition to making long hours he attributes his success to the richness of the phenotypes and diseases available in the data at his disposal. Regarding his high number of co-authorships, he argues that it is not problematic, but rather reflects the sheer magnitude of the network and effort required to achieve these types of scientific discoveries (Supp Mat)^32^. A third group of authors are individuals who have led multiple key consortiums (e.g., CHARGE) focused on prominent traits such as obesity, type 2 diabetes and cardiovascular disease. Their high GWAS *H*-Index comes in part from their ability to contribute the same data sets to examinations of multiple traits and renewed rounds of study on the same trait which incorporate larger and larger sample sizes. Nine of the top 10 researchers are based at European institutions (and Albert Hofman was at the Erasmus Medical Center, Netherlands until 2016).

**Table 4:** The top 10 most prominent GWAS authors

Name author	N-papers	Citation count	GWAS H-index	Network betweenness	Network centrality	Country	Institution
Kári Stefánsson	177	27568	84	0.020	0.308	Iceland	deCODE genetics
Unnur þorsteinsdóttir	142	23633	77	0.006	0.241	Iceland	deCODE genetics
Albert Hofman	267	25534	76	0.013	0.345	U.S.	University of Harvard
André G. Uitterlinden	280	23337	76	0.018	0.367	Netherlands	Erasmus MC
Cornelia M van Duijn	188	20879	71	0.008	0.294	Netherlands	Erasmus MC
Gudmar Thorleifsson	119	20408	70	0.006	0.232	Iceland	deCODE Genetics
Christian Gieger	166	22562	70	0.011	0.272	Germany	Helmholtz Zentrum München
Panos Deloukas	109	20323	68	0.009	0.233	U.K.	Queen Mary University of London
H-Erich Wichmann	112	20266	68	0.007	0.220	Germany	Helmholtz Zentrum München
Fernando Rivadeneira	198	17976	65	0.009	0.282	Netherlands	Erasmus MC

Automated and manual (web search) curation of details regarding authors ranked within the 10 highest GWAS H-Index (an estimate of the importance, significance, and broad impact of a scientist’s cumulative GWAS-related research contributions). N-Papers refer to the number of times the author features as an author (at any position) within the Catalog. Information on citations comes from PubMed Central. Betweenness and Degree centrality calculated with Network-X. All characters converted to ASCII to ensure maximum matches of the same authors across papers

We also examined the most frequently returned Consortiums (termed Collectives in the PubMed database). Of all unique PubMed IDs queried, 844 refer to at least one consortium, with an estimated total of 1654 contributions from 681 unique consortia. The top five consortiums ordered by the number of (cleaned and harmonized) returns are: Wellcome Trust Case Control Consortium (49 returns), CHARGE (46), Wellcome Trust Case Control Consortium 2 (36), the LifeLines Cohort Study (30), and DIAGRAM (29).

In Table 4, only two of the 10 senior authors are female, leading us to explore different aspects of gender imbalance. A growing number of studies have flagged gender imbalance in scientific publications and funding^8,33,34^. We estimate that men contribute 63.03% of all authorships and represent 59.62% of all unique authors. This allows us to naively infer that men contribute more papers on average (per author) than women. These results are best examined in the context of recent work^35,36^ based on the entire JSTOR corpus, which estimates that 27.27% of academic authorships between 1990 and 2011 are on aggregate female. This figure increases to 29.3% when filtering for authorships in the field of Molecular and Cell Biology (and to 32.4% for the specific subdiscipline of Human Genomics). Our estimate of 36.97% is higher than these figures, and even more so when compared with the historical average of women undertaking research in Molecular and Cell Biology (20.7% between 1665 and 1989).

We build on work showing the historical under-representation of women in the first and last authorship positions^36–40^. Our analysis shows that 44.04% of the authors in the first author or junior position are female: substantially higher than the all positions estimate. This decreases to just 29.66% for authorships in the senior last author position: substantially lower than the all positions estimate (albeit still higher than other estimates spanning 1990–2011 in the Human Genome subdiscipline^36^) or first authors of commentaries in *Nature* (20.0% in 2016)^33^. This is potentially owig to a historic gender imbalance in educational attainment in scientific fields, with fewer women obtaining doctorates in the past than today. We found similar average GWAS-Indexes for female (4.85) and male (5.34) authors and compared the average number of papers published by females (6.15) and males (7.17) and the average number of citations (648.44 for females, 780.73 for males). Finally, we examined whether there are gender differences across the most frequently studied EFO terms. Here, we find a striking concentration of female authorship in studies of Breast Carcinoma (51.0%), whereas male authors are concentrated on Schizophrenia and Type 2 Diabetes with only 31.0% and 33.0% female authorships, respectively (with these numbers almost wholly invariant as to how we split the EFO terms in the Catalog).

## Funders: US and UK dominate

To understand geographic concentration, we examined the funding sources of research. Using the PubMed database, we find a total of 136 different funding agencies, with funding being spread across a total of 12,790 unique Grant IDs. Each study has an average of 13.52 contributing grants. We measure each unique grant contributing to one study (PubMed ID) as one acknowledgment with the five most frequently acknowledged funders being: NHLBI NIH HHS (National Heart, Lung & Blood Institute; 25.88%), NCI NIH HHS (National Cancer Institute; 10.64%), NIA NIH HHS (National Institute on Aging; 8.37%), MRC UK (Medical Research Council; 7.21%), and NIMH NIH HHS (National Institute of Mental Health; 5.50%). The most commonly acknowledged single grant (P30 DK063491, 207 times) is from the National Institute of Diabetes and Digestive and Kidney Diseases (NIDDK) and is a Core Centre Grant supporting the UCSD/UCLA NIDDK Diabetes Research Center.

Most of the funding acknowledgments are to US agencies (85.11%) and primarily relate to programs funded by the NIH (apart from the Public Health Service). This is followed by the UK (14.37% of total), with a high number of acknowledgments not just to the MRC, but also to the Wellcome Trust (3.73%), and Cancer Research UK (1.23% of total). This contrasts with other returned countries including: Canada (0.36%), ‘International’ (0.14%), Austria (0.01%), and Italy (0.01%).

**Fig. 4:**
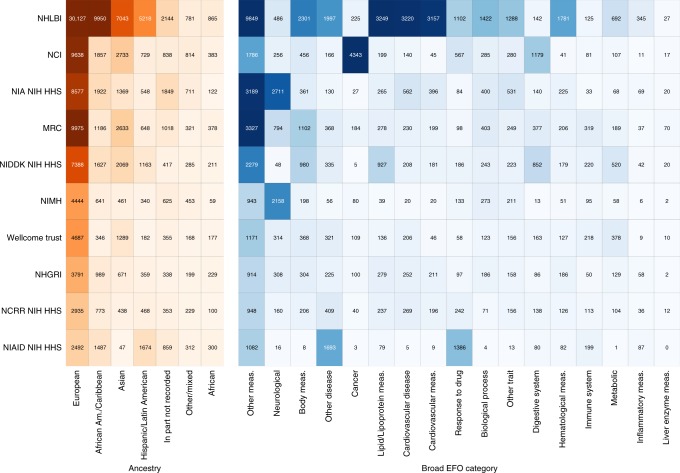
Distribution of Funder Acknowledgments by Ancestry and Trait Categories. Heatmap showing the distribution of Grant Contributions of the 10 most frequently observed agencies tabulated against our synthetic broader ancestral category term and Parent Term fields (higher level trait or disease categories). All agencies are based in the US, other than the Medical Research Council (MRC) and Wellcome Trust. In the US, other than Public Health Service, the rest are part of the National Institute of Health (NIH). Replication material provides an alternative mapping to Broad EFO category where comma separated entries are not split but dropped. Source: NHGRI-EBI GWAS Catalog and the PubMed database

We also summarize the broad ancestral patterns and the distribution across broad disease categories studied when tabulated across various funding agencies in Fig. 4. The NIH Revitalization Act of 1993 (Subtitle B, Part I)^41^ implemented a policy regarding the inclusion of minorities as subjects in clinical research (where a minority is defined as a readily identifiable subset of the US population that is distinguishable by racial or cultural heritage)^42^. The Medical Research Council (the largest UK funder) has no similar restriction, although one fund that forms part of the UK’s Wellcome Trust solicits proposals, that promote diversity and inclusion, and engages people and communities who are affected by social and economic disadvantage^43^. An important feature of the figure is the comparatively lower ratio of European to non-European ancestries in NIH-funded research in comparison with UK-funded research, which is not legislated to diversify participants. In terms of traits, we see the expected clustering around terms corresponding to the missions of each respective funder. For example, the most frequently funded term from the National Cancer Institute (NCI) is Cancer.

## Future directions

### Recommendation One: prioritize the inclusion of multiple types of diversity

These findings lead us to 10 evidence-based policy recommendations. Recommendation One is that researchers, editors, funders, and commercial companies prioritize the inclusion of multiple types of diversity in data, namely: ancestral, geographical, environmental, temporal and demographic, and recognize the impact that this lack of diversity has on research findings. First, ancestral diversity needs to increase beyond the replication phase to include more non-European ancestry populations. Significantly extending previous comparisons^22^, we show that diversity levels fluctuated markedly. Following the full release of the UK Biobank and increased reliance on large direct-to-consumer data, we predict that diversity in GWAS ancestry may decrease even further, given that 94.23% of the 488,377-genotyped UK Biobank participants are in the white ethnic group^44^ and 23andMe has a sample with 77% European ancestry^45^.

The benefits of increased ancestral diversity are multiple; GWAS that utilize data from diverse populations will provide more accurately targeted therapeutic treatments to more of the world’s population, extend insights into the architecture of traits and uncover rare variants with significant effect sizes, which replicate across ancestries. Isolated populations–owing to bottleneck events, genetic drift, adaptation, and selection–are of importance owing to higher frequencies of rare variants, which increase the power to detect associations with clinically important phenotypes^46^. Discovery is often boosted in populations with high rates of homozygosity such as those with a tradition of consanguineous marriage. A recent study of exomes of British Pakistani adults with high parental relatedness, for instance, discovered rare-variant homozygous genotypes that predicted “knockouts” (loss of gene function) in hundreds of genes^47^.

Although the focus has primarily been on increasing ancestral diversity, we also call for an expansion of both geographical and environmental diversity. Although ~ 76.2% of the current world population reside in Asia or Africa^48^, we estimate that 72% of genetic discoveries emanate from participants recruited from only three countries (US, UK, Iceland). By examining only genotype–phenotype associations, GWAS have largely ignored the fact that complex traits have a strong geographical component involving genetic predisposition and environmental exposure. There is little reflection on how environmental variation or Gene–Environment (G×E) interaction impacts results or even shapes the traits that are prioritized for research^49^. The US, UK, and Iceland have distinct histories and social systems that have fundamentally shaped exposure to certain disease factors or traits. Those predisposed to obesity for instance, face radically different environmental stimuli in the US than in other nations. Or, those with a higher genetic predisposition to skin cancer would have their risk exacerbated if they resided in areas with higher sunlight exposure. GWAS regularly combine data sets from vastly different countries and historical periods with little recognition of the consequences, implicitly assuming the impact of genetic loci on traits is universal across time and place. A recent study shows that for complex traits, a large proportion of genetic effects are hidden or watered-down when disparate data across different countries and historical periods are combined^50^.

We also advocate an increased temporal diversity of individuals across different birth cohorts, historical periods and life-course stages. We estimate that the most frequently used data sets are disproportionately populated by older individuals, yet the prevalence and measurement of disease varies considerably with age. There is only a moderate positive correlation between midlife and old-age measures for body mass index, glucose, and systolic blood pressure, for instance, which all increase with age^51^. Samples of older individuals also suffer from mortality selection and exclude a non-random subset of the population^52^. This issue is compounded by healthy volunteer selection and participants with a high socioeconomic status, both of which occur disproportionately in prominent large data sets such as the UK Biobank^28^. Finally, we call for more discussion related to the gender diversity of GWAS participants, particularly regarding specific diseases as there is growing evidence of sexual dimorphism in traits linked to obesity^29^, reproduction^30,53^, and others.

### Recommendation Two: monitoring with funding consequences

Beyond policy formation regarding diversity or gaps in research to intensive monitoring with consequences for funding. Our scientometric approach that links funders, researchers, and grant IDs to ancestral and geographical coverage provides a cost-effective first step toward transparent monitoring in this direction with the potential to expand and locate knowledge gaps in research into certain clinical traits.

### Recommendation Three: careful interpretation of genetic differences

European ancestry-based polygenic scores derived from GWAS explain only half as much of the variability in the phenotype for non-Hispanic Black samples as compared with non-Hispanic Whites^20,54^ and many cancer associations fail to replicate in other populations^55^. There is a danger that the inability to apply polygenic scores from European ancestry studies to other groups is misinterpreted to reflect biological differences between different ethnic or racial groups. This misnomer was carefully discussed, for instance, in a recent GWAS of educational attainment^56^. Genetic variation needs to be distinguished from the social, cultural, and political meanings ascribed to different human groups^57,58^. Race is not a biological category, as genetic variation is traced to geographical locations and does not map into our perpetually evolving and socially defined racial or ethnic groups. Dictionary-based exercises herein have revealed categorizations that often combined geographical, migration, and ancestral background. Populations are the product of repeated mixtures over tens of thousands of years^20^. Although we use the dominant broad ancestral categories common in the field, by noting these issues we recognize that a more sophisticated categorization scheme is required.

### Recommendation Four: local participant and researcher involvement

Previous research has noted lack of local participant and researcher involvement when collecting genetic material in underrepresented communities^57,59^. There are encouraging endeavors to increase genotyping outside of North America and Europe such as the African Genome Variation Project^60^. Many projects that collect non-European samples have funding from large research bodies such as the NIH or Wellcome Trust, granted primarily to researchers working in those countries. The danger, however, is that helicopter science—collecting and then exporting genetic data—may compound existing inequalities, with participants and researchers from those countries not being the main benefactors. African researchers have recently noted that many have accepted restrictive terms offered by foreign partners owing to a lack of resources to handle large genomic data sets^61^. We recommend the inclusion of meaningful local intellectual contributions and, if required (in addition to data collection), the supply of training, computational resources, and infrastructure development to enable local scientists to build the capacity to work independently.

### Recommendation Five: action to reduce inequalities in authorship and investigators

We estimate that women author on average fewer GWAS papers, have fewer citations than men, are more frequently junior first authors and less frequently senior authors. The latter observation is remarkably similar to NIH figures, where women constitute only 30% of principal investigators on grants^62^. This suggests a relationship between acting as a senior author and functioning as a PI on grants and may contribute to women’s lower peer review scores on funding panels^8^. The NIH has established initiatives such as the Women in Biomedical Careers Working Group and the 2017 Next Generation Research Initiative. Policies such as these which target early career researchers are more likely to reach this goal since these groups are more often more ethnically diverse and populated by a higher percent of women^9^. Female researchers themselves need to be cognizant of these disparities, as should those who conduct research appraisals and funding reviews.

We were unable to control for maternity or care leaves, which may have a role in productivity and serving as a PI, particularly in some European countries where women may take up to 1 year leave^63^. This echoes recent findings that women had a lower longevity in funding, witnessed by a lower likelihood to renew projects, lower submission rates, and lower funding per year^8^. Women face distinct work-life reconciliation issues and may require additional mentoring and support to encourage them to submit and renew applications or serve as a PI. Increased gender diversity in science may also lead to fundamentally new discoveries. That can have real clinical consequences: consider for instance that symptoms of cardiac arrest in women were ignored and misdiagnosed for decades. This has been attributed to the notion that coronary disease was considered a male only health concern, largely studied in male subjects by male scientists.

### Recommendation Six: reform incentive structures that intertwine the role of authorship, data ownership, and dating sharing

GWAS demand collaboration through the formation of large consortiums, resulting in multiple authorships. As illustrated (Fig. 1. and Fig. 2), large samples are required owing to the relatively small effect sizes, with the number of detected associations typically increasing with sample size. Central authors within the GWAS network are the holders of large longitudinal data sets or those who lead large consortiums, with many top GWAS scientists classified as hyperprolific^32^. We reinforce the necessity of conventions related to author transparency in contributions, such as via the Vancouver Regulations which describe the contributions of individual authors^32^. With hundreds of authors, full transparency and reporting remains a challenge. A related suggestion could be to distinguish between authors and contributors who provide data. Another could be to provide data producers with a 6–12-month grace period before making data publicly available to similarly interested researchers. This, however, has the potential to generate its own incentive-based anomalies and pressures.

These solutions, however, do not align with current incentive and reward structures. When the PI and participating researchers are evaluated, it occurs at the individual level. In the UK’s national Research Excellence Framework (which ranks departments and institutions according to research excellence), for instance, authorship is a key return. To remove individuals from GWAS authorship demands a broader discussion of incentive systems applicable to data generators. Some observers argue that the authorships of scientists who obtained the funding, designed the study, supervised staff and students, and often supervise data collection and analyses should be removed. Yet, without such labor-intensive endeavors, GWAS would not exist. We also call for the careful application of research metrics such as the H-Index, particularly when comparing scientists and academics across scientific disciplines. As a leading GWAS author and holder of one of the most used GWAS data sets carefully warns: “…for comparing these authorships across different scientific disciplines (biomedical and beyond) I think we should revisit this issue with a critical appraisal to create a better understanding among fellow scientists”. (p. 104 Supp Mat)^32^.

### Recommendation Seven: create digital object identifiers (DOIs) for data sets and enforce ORCID iDs for authors

An implicit part of this, related to Recommendation Six, is the invitation to publish Data Resource style articles, which generate DOIs for each data source to reward data collection. Surprisingly, our manual curation of data sets revealed a striking lack of transparency and inconsistency in describing the basic data source or additional sample restrictions utilized in many papers. Even in the most eminent journals, descriptions of data were cryptic and sources unclear or untraceable, raising issues of transparency and reproducibility of research. The opening of publicly funded databases has enabled this review to take place, and newly emerging Application Programming Interfaces represent just one small part of the sweeping advancements. However, the implementation of DOIs for common data sets, and the encouraged use of ORCID iDs for authors—in the same way that PubMed IDs identify papers and EFO terms represent experimental variables—would enable better scientometrics and a more accurate reflection of genomic science.

### Recommendation Eight: coordinated governance from multiple stakeholders

There have been repeated calls to remove barriers and increase trans-border cooperation, such as UNESCO’s reiteration that it is a human right to benefit from shared scientific advancements^64^. There are striking differences in national regulations for data sharing and a patchwork of Institutional Review Board (IRB) positions. International models of genomic data sharing do exist, such as those pioneered by the International Cancer Genome Consortium. A recent evaluation of genomics data sharing across multiple countries reveals complexity, contradiction, and confusion^64^. Data transfer to third countries outside of China, for instance, is prohibitive owing to overlapping and complex data regulations. The US has a fragmented data protection regime with oversight across IRBs and data access committees^65^. Europe’s recent General Data Protection Regulation (GDPR) brought new restrictions related to the transfer of data across borders, complicated by additional unique country–and institutional–specific interpretations^66^. An international genomics group could create a more transparent code of conduct and shape the interpretation of GDPR’s rules. Closely related to this is the further development of the regulatory protection and data sharing across borders in relation to cloud based storage providers. Those who store the data are dependent on cloud providers who often shift data across geographical locations with limited notification or oversight^67^.

### Recommendation Nine: enforce the sharing of GWAS summary results

Just as data can serve as a valuable commodity, so can summary results. Although such sharing is a requirement of many major journals, it remains a policy gray area and they are regularly not released, even after directly contacting authors. Others share only when co-authorship is granted. An effective deterrent could be the threat of retraction of the article unless summary results are shared or prohibiting applications or granting future funding until past discoveries are made publicly available.

### Recommendation Ten: utilize influence for the good of more people

Our last recommendation highlights the fact that data sharing, ethics, and transparency is frequently discussed with the implicit assumption that funders, ethics boards, and universities are the only bodies with the power to govern this ecosystem. But what if researchers do not need funding or operate outside of universities and their incentive systems? The growth of direct-to-consumer companies such as 23andMe and biomedical companies, many of whom hold the largest genomic data sets, often fall outside of regulations of funders or universities. By virtue of their position, data sharing, and release of results often follow different rules than publicly funded data sets. Some impose the restricted release of GWAS summary statistics (i.e., the information that is used by other researchers to create polygenic scores and additional analyses). Considering the recent sales of blocks of direct-to-consumer data to pharmaceutical companies^68^, scientific collaboration also has the potential to be restricted. Although commercial genomics companies generally operate with different demands and incentive structures, most still require external validation of their results published in top scientific journals, placing editors, and journals in a key position of power. We conclude thus by calling upon all parties in the genomics ecosystem to utilize their influence for the good of more people as part of the ongoing genomic revolution.

### Conclusions

Our systematic scientometric review of genomic discovery quantifies multiple known and unknown assumptions about this domain. We observe considerable fluctuation in the ancestral diversity of participants over time. By ranking the most frequently used data sets, we also went beyond ancestral diversity to show other types of selectivity. We mapped the geographical recruitment of GWAS participants and core funders by ancestry and disease coverage, explored gender disparities in authorship and provided evidence of a tightly knit social network of researchers and consortiums. A central finding was that our results once again emphasized the potential for a cycle of disadvantage for underrepresented communities and despite continued efforts, infusing diversity into genomics remains challenging.

### Code availability

A full standalone GitHub repository (github.com/crahal/GWASReview), which predominantly runs off a Jupyter Notebook and supporting functions accompanies this article as Replication Material. This repository also contains the latest versions of all outputs discussed in the text, in terms of full lists of author rankings, funder acknowledgments, and so forth. The generalized code will enable clones of the repository to provide dynamic advancements over time.

## Supplementary information

Supplementary Information
