# Supplementary material for: A scientometric review of genome-wide association studies
Source: Commun Biol. 2019 Jan 7;2:9. doi: 10.1038/s42003-018-0261-x (PMC6323052; doi:10.1038/s42003-018-0261-x)
Supplement: Supplementary file 1 — Supplementary Information [file 42003_2018_261_MOESM1_ESM.docx]

A Scientometric Review of Genome-Wide Association Studies

**Supplementary Material**

***Communications Biology***

Melinda C. Mills^[[1]](#footnote-2)^* (ORCID: 0000-0003-1704-0001)

Charles Rahal (ORCID: 0000-0002-1764-2697)

University of Oxford and Nuffield College

Supplementary Note 1

Replication Material

This document describes the materials and methods used in the paper. In addition to the description of Materials and Methods (the Supplementary Material), we also include online Replication Material of all code and data used in this study to allow reproducibility of results and further extensions. This is hosted as a standalone GitHub repository (github.com/crahal/GWASReview) which predominantly runs off a Jupyter Notebook and supporting functions. This repository also contains the latest versions of all outputs discussed in the text, including full lists of author rankings, funder acknowledgements and so forth.

Supplementary Methods

Materials

Four main data sources were used for this article. First, we draw on over thirteen years of GWAS discoveries (March 2005 to October 2018) from the NHGRI-EBI GWAS Catalog (hereafter ‘the Catalog’) produced by the US National Human Genome Research Institute (NHGRI) in conjunction with the European Bioinformatics Institute (EBI).(*1*, *2*) The Catalog is regularly updated with all new English-language GWAS studies curated from PubMed, with a significant degree of quality control by experienced molecular biologists involving multiple rounds of data extraction and validation before indexation.(*2*) Eligible studies are typically indexed within 1-2 months of publication, although the lag time between studies varies. While the code is dynamic and can produce updated analysis with minimal outlay, this article relies on the 2018-10-29 editions of three main files from the Catalog; ‘All Studies (v.1.0.2)’, ‘All ancestry data’ and ‘All associations v1.0’.

We link this to the ‘GWAS to EFO Mappings’ file (Experimental Factor Ontology: a combination of several biological ontologies used by the EBI) which is then merged to the main files via the ‘EFO term’ and ‘MAPPED_TRAIT’ fields of the Catalog. The Catalog is then linked to PubMed and PubMed Central via a range of Entrez Programming Utilities. Our fourth and final sources of data are contextual databases. To undertake our geographic analysis of participant recruitment, we use shapefiles from Natural Earth (v4.0.0) and subsequently utilize the attribute fields which correspond to the CIA World Factbook to merge the Catalog's (cleaned) country of recruitment field to sovereign states and corresponding continents. The linked population estimates are based on the 2017 World Population Prospects by the United Nations.(*3*)

In addition to the computational approach described above, an extensive manual data curation exercise involved the extraction, coding and harmonization of the names of all cohort datasets used in approximately the largest 1,250 GWAS papers indexed by the end of July 2018, grouped by PubMed identifier (covering over 85% of all observations across papers). This is augmented with data from published cohort descriptions and direct correspondence with cohort leaders and administrators. This allowed us to create a list of the most frequently used cohort data across all GWAS, merging sub-cohorts where appropriate into their respective projects (e.g. RS-I, RS-II and RS-III map to the larger Rotterdam Study (RS)).

**Methods**

We utilize Python 3.6, a popular interpreted high-level language for general-purpose programming. We conduct all analyses using the IPython kernel for a Jupyter Notebook which accompanies this paper (and is simultaneously hosted on GitHub). This is in addition to a number of ‘support functions’ which call APIs, clean data, conduct analysis, and so forth. Notable libraries used include matplotlib, pandas, NetworkX (to calculate ‘Betweenness’ and ‘Degree’ centrality measures in the network analysis) and BioPython (for the Entrez Programming Utilities). The accompanying repository contains the necessary details of dependencies for replication and is described in the corresponding readme file.

To examine ancestry, we only use data from the Catalog. The Catalog’s own ‘broad’ ancestry field is based on a manual mapping of the free text ancestry description. For example, ‘Han Chinese’ maps to ‘East Asian’ in the ‘broad’ field, either uniquely or with other ancestry combinations. We then map all these combinations of the seventeen different ancestral categories to seven unique ‘Broader’ categories (for example, ‘East Asian’, ‘South Asian’ and ‘Asian (NR)’ all map to ‘Asian’) using a custom, manually curated dictionary. To consider polyvocality and native/indigenous populations, we design functions primarily based on regular expressions to exploit patterns in the "INITIAL SAMPLE SIZE" and "REPLICATION SAMPLE SIZE" free-text fields. For example, our function cleans, corrects for common spelling errors and re-structures the text of “19,546 Europen ancestry individuals from 6,863 families” into two separate fields of “19,546” and “European” which can then be used for further downstream analysis.

To produce our choropleth map of country of recruitment, we only utilize values in the ‘Country of Recruitment’ Catalog field when only one country is mentioned. As this field does not contain a unique mapping to Study Accession (meaning we cannot automatically ascertain the sample size from each country for every study), we drop studies which recruit from multiple countries. For example, if N=100,000 and Country of Recruitment is ‘U.K., U.S.’, there is no way for us to decipher the breakdown between the two countries in the Catalogue (especially as the free text field may just contain 'European' ancestry). We also drop studies for which the country is ‘Not Recorded’ (NR). This leaves us with a total of 5,348 entries which we then clean (e.g., 'Republic of Korea' to 'Korea, South') to merge to a list of sovereign states (and corresponding continents) recognized by the CIA’s World FactBook.

To consider areas of study (‘Parent Terms’), we link the Catalog to the ‘GWAS to EFO Mappings’ file via the ‘EFO’ term and ‘MAPPED_TRAIT’ fields of the Catalog. To overcome the issue of multiple (comma separated) mapped traits occurring within around a third of the Catalog’s rows, we split rows with multiple traits and a single Accession into multiple rows. For instance, a recent entry in the Catalog (PubMed ID 29330379) with this feature examines the shared genetic variants between schizophrenia and lung cancer. We view this analytical strategy as logical and preferable to dropping rows with multiple traits and nor do our results significantly change relative to when we do so. The replication files produce estimates and figures where these rows are split and when they are dropped.

We then link the Catalog’s PUBMEDID field with data on acknowledged funders, authors, consortia and citations. To measure the impact of authors in the field, we derive a new ‘GWAS *H*-Index’, which is analogous to a classic *H*-index; an estimate of the importance, significance and broad impact of a scientist’s cumulative research contributions.(*4*) We then infer gender based on a methodology similar to other approaches in the academic literature,(*5*) but more specifically based on the gender.c files of Michael Jörg. When doing so, we filter out initials and focus on complete first names only, dropping returns that indicate that a name might be considered androgynous or where the gender cannot be inferred with confidence based on traditional naming conventions. When analyzing the gender divide across authorship position, we focus on papers with more than four authors. In our social network analysis, the measure of degree centrality assigns a score of relative importance based on the links held by each author to other authors (utilizing co-authorship relations as edges) and betweenness centrality measures the number of times an author lies on the shortest path between other co-authors. We calculate centrality measures based on a network of authors with a minimum of one paper and ten citations. While we envisage it will be possible to soon analyze across ORCID records, this is currently intractable, and we are restricted to unique forename, initial and surname combinations to create a unique identifier. For analysis of consortia, we manually curate a dictionary to harmonize the PubMed returns, where, for example ‘AGEN’ and ‘AGEN Consortium’ are mapped to the same entity.

Supplementary References

1. D. Welter *et al.*, The NHGRI GWAS Catalog, a curated resource of SNP-trait associations. *Nucleic Acids Res.* **42**, 1001–1006 (2014).

2. J. MacArthur *et al.*, The new NHGRI-EBI Catalog of published genome-wide association studies (GWAS Catalog). *Nucleic Acids Res.* **45**, D896–D901 (2017).

3. UN-DESA, “Total Population - Both Sexes, 2017” (2017).

4. J. E. Hirsch, An index to quantify an individual’s scientific research output. **102**, 16569–16572 (2005).

5. J. D. West, J. Jacquet, M. M. King, S. J. Correll, C. T. Bergstrom, Gender composition of scholarly publications (1665 - 2011) (2013), (available at http://www.eigenfactor.org/gender/#).

1. *Correspondence: Melinda C. Mills, Nuffield College, New Road, Oxford, OX1 1NF, UK Email: melinda.mills@nuffield.ox.ac.uk. Tel: +44 1865 278500. Twitter: @melindacmills. Supplementary replication material which can dynamically update the analysis over time can be found at https://github.com/crahal/GWASReview. [↑](#footnote-ref-2)
